# Lipid lowering therapy patterns and the risk of cardiovascular events in the 1-year after acute myocardial infarction in United Arab Emirates

**DOI:** 10.1371/journal.pone.0268709

**Published:** 2022-09-02

**Authors:** Lionel Pinto, Mohamed Farghaly, Sasikiran Nunna, Badarinath Chickballapur Ramachandrachar, Sri Harshadeep Chilukuri, Ashok Natarajan

**Affiliations:** 1 Amgen Inc., One Amgen Drive, Thousand Oaks, California, United States of America; 2 Dubai Health Authority, Dubai, United Arab Emirates; 3 IQVIA, Plymouth Meeting, Pennsylvania, United States of America; 4 IQVIA, Dubai World Trade Centre, Dubai, United Arab Emirates; Medizinische Universitat Graz, AUSTRIA

## Abstract

**Aim:**

In United Arab Emirates, cardiovascular disease (CVD) is a leading cause of mortality and 22% of CVD deaths are attributable to acute myocardial infarction (MI). Adherence to guidelines for lipid management is incompletely described in the Middle East. This study aimed to characterize lipid lowering therapy (LLT) patterns and the risk of subsequent cardiovascular events (CVEs) in the first year after MI.

**Methods:**

This was a retrospective cohort study using the Dubai Real-World Claims Database, including all patients discharged with MI between January 01, 2015 and December 31, 2018, followed-up until December 31, 2019.

**Results:**

In the first year after MI, 8.42% of 4,595 patients included experienced at least one recurrent MI (rate 6.77 events/100 person-years [PYs]), 2.94% had one revascularization (cumulative rate 0.55 events/100 PYs) and 2.66% had one hospitalization due to unstable angina (cumulative rate 5.16 new events/100 PYs). The majority (60.40%) of the patients presented with LDL-C levels ≥ 70 mg/dL after MI. In the first year after MI, 93.45% of the patients received LLT, mainly high-intensity statin (67.79%); with a minority of patients receiving statin + ezetimibe (4.55%), PCSK9i (0.20%) or ezetimibe alone (0.07%).

**Conclusion:**

Patients hospitalized with MI in Dubai present an increased risk of CVEs in their first-year post-discharge. Majority of the patients presented with LDL-C levels above 70 mg/dL, which indicates suboptimal lipid control with existing LLT, particularly in high-risk patients.

## Introduction

Cardiovascular disease (CVD) and the associated burden are increasing globally and represent a key challenge in healthcare. The World Health Organization has reported that CVD is the primary cause of death worldwide, accounting for 17.9 million deaths (31% of all deaths) in 2016. Over three-quarters of CVD deaths occur in low and middle‑income countries [1]. The Middle East has been reported to have the highest increasing CVD-associated mortality rate in the world [2, 3]. In United Arab Emirates (UAE), CVD is a leading cause of mortality and of CVD deaths, 22% were attributable to acute myocardial infarction (MI), 16% to cerebrovascular disease, 6% to ischemic heart disease and 5% to hypertension [4]. Moreover, the Gulf Registry of Acute Coronary Events and its second iteration (Gulf RACE and Gulf RACE-2) demonstrated that patients with acute coronary syndrome in the Arab Middle East are younger than in developed countries and have higher rates of diabetes and smoking [5, 6].

Survivors of MI are at high-risk of cardiovascular events (CVEs) such as stroke, recurrent MI or cardiovascular death, and studies have shown this risk is higher in the first year following the index MI [7, 8]. These findings reinforce the importance of both acute clinical care and secondary prevention in improving outcomes for patients with MI. Numerous studies have highlighted the importance of lowering cholesterol, specifically low-density lipoprotein cholesterol (LDL-C) in patients with cardiovascular risk [9–11]. Although international guidelines for managing plasma lipids exist and there is agreement on most of the key recommendations, there is, however, a lack of awareness and adherence to these guidelines by local healthcare professionals in the Middle East [2, 12]. In the Africa Middle East Cardiovascular Epidemiological cross-sectional study, UAE was one of the top 5 countries with the highest prevalence of dyslipidemia (exceeding 70%) [13].

Therefore, using the Dubai Real-World Claims Database, the current study aimed to characterize the risk of subsequent CVEs in survivors of MI during the first year after index MI discharge and lipid lowering therapy (LLT) patterns (primary objective), and describe their subsequent LDL-C levels (secondary objective). The findings of this study can contribute to a better understanding of the clinical management of MI in real-world clinical practice in UAE, providing valuable evidence to inform primary and secondary prevention of CVD in this region.

## Methods

This was a retrospective cohort study using the Dubai Real-World Claims Database. This database is an anonymized longitudinal patient level database of insurance claims generated from the private healthcare sector in the Emirate of Dubai. An ethics committee approval was not required for the analysis of this anonymized retrospective patient dataset. Less than 0.1% of the claims in the dataset are from the public sector. The database comprises over 10 million patients who are UAE residents and have claims for treatment from a medical facility located in Dubai. It contains information on patient demographics, diagnoses, procedures (medical, surgical, and diagnostic), prescriptions, and other related services. The database captures 100% of the population covered by private health insurance in Dubai. As, approximately 80% of the population in Dubai is covered by private insurance (predominantly comprising the expatriate community) while the remaining 20% are covered by public funding (comprising the local Emirati population), the Dubai electronic (e)-Claims are representative of the multi-ethnic population of Dubai.

This study included all adult patients with atherosclerotic cardiovascular disease (ASCVD) registered in the database. To be included in the analysis of the primary objective, patients had to fulfill all the following criteria: 1) Patients with an index event (MI) between 01st January 2015 and 31st December 2018 (study inclusion period); 2) Patients aged ≥18 years at index date; 3) Patients with continuous enrollment (Patients with at least one claim for any service (MI or Non-MI) during the 6-months in the pre-index period and during the 6-month in post-index period), as this criterion is a surrogate for ensuring the inclusion of patients who are registered and active with their medical practice. Patients with missing age, gender and other data quality issues were excluded from the study. To be included in the analysis of the secondary objectives, patients also had to have at least one LDL-C measurement 6-month post-index date.

*MI cases were ascertained from the database using* inpatient medical claims and procedure *codes*, *and the date of the first MI record within the inclusion period was termed as the index date*. The overall study period (January 2014 to December 2019) allowed at least 12-months of data pre-index period (Baseline period) and at least 12-months data post-index period (follow-up period) for all patients. See Supplementary Materials for further details on the methodology used (including code lists used [S1 Appendix], data transformation definitions [S2 Appendix] and handling of missing data [S3 Appendix]).

The statistical analyses were mainly descriptive. Continuous variables were summarized using standard summary statistics such as number of observations, mean and median values, standard deviation (SD). Categorical variables were summarized in frequency tables as counts and percentages of the total study population, and by subgroups where appropriate. Cumulative post-index CVE rate per 100 person-years (PYs) was calculated for all CVE types (MI, ischemic stroke [IS], unstable angina [UA], revascularization, composite of MI or IS and composite of all acute CVEs), from the index date to the end of one-year in the post-index period, using the following formula:

$$\frac{Number\;of\;distinct\;CV\;events\;in\;all\;post\;–\;index\;period\;*\;100}{\begin{array}{l} Patient-years\;from\;index\;to\;the\;earliest\;of\;the\;following:\\ \;End\;of\;reporting\;period\;(1\;yr)\\ \;End\;of\;the\;Continuous\;Enrollment\;(CE)\\ \;End\;of\;study\;period\;(December\;31,\;2019) \end{array}}$$

## Results

Table 1 presents a summary of the selection of patients to be included in this study, according to the pre-specified inclusion and exclusion criteria. A total of 4,595 patients were included in the final analysis (primary objective) and 1,740 patients were included in the analysis of post-index LDL-C levels (secondary objective).

**Table 1:** Study sample selection.

	Number of Patients			
	Included		Excluded	
	N	%	N	%
**Inclusion Criteria **				
1) One claim with MI diagnosis during the index period (01 January 2015 to 31 December 2018)	7,904	100%		
2) One claim for any service 6-months pre-index date and one claim 6-month post-index date (surrogate of CE)	5,571	70.5%	2,333	29.5%
3) Age ≥18 years at index date*	5,499	69.6%	72	0.9%
**Exclusion Criteria **				
4) Missing age, gender and other data quality issues	4,595	58.1%	904	11.4%
**Patients in the final sample for primary objective**	**4,595**	**100.0%**		
5) One LDL-C measurement 6-month pre-index date	3,523	76.7%	1,072	23.3%
**6) One LDL-C measurement 6-month post-index date (sample for secondary objective)**	**1,740**	**37.9%**	2,855	62.1%
7) One LDL-C measurement during both the 6-month post- and pre-index date	1,404	30.6%	3,191	69.4%

CE–Continuous Enrollment; LDL-C—Low-density Lipoprotein Cholesterol.

*latest age available as per the claims database and age imputation considered for patients with missing demographic details.

At discharge from MI hospitalization, the mean age of the patients was 52.4 years (SD 12.5 years), with the majority being male (N = 3,865; 84.11%). In the 12-months before index MI, 88.05% (N = 4,046) of the patients received LLT, mainly statins (N = 3,934; 85.61%) and those of high-intensity (N = 3,163; 68.84%) (see S1 Table for details on the classification of statins therapy intensity). A minority of patients received a combination of statin + ezetimibe (N = 108; 2.35%), 2 patients received PCSK9i and 4 patients received ezetimibe alone (Table 2). A similar pattern of LLT use was also observed in the 90 days before MI as shown in S2 Table. In this baseline period, the most frequent ASCVD diagnoses were coronary revascularization (Percutaneous Coronary Intervention; PCI) (N = 985; 21.44%) and acute coronary syndrome (MI or UA) (N = 616; 13.41%). Most patients presented comorbid cardiovascular risk factors, such as hypertension (N = 2,959; 64.40%) and diabetes (N = 2,055; 44.72%). Similar baseline characteristics were observed for the 1,740 patients included in the analysis of the secondary objective. In the 6-months before index MI, patients presented with average LDL-C levels of 142.6 mg/dL (SD 61.4 mg/dL), with the majority presenting with LDL-C levels ≥ 70 mg/dL (N = 1,303/1,404; 92.81%) (Table 2).

**Table 2:** Baseline clinical and treatment characteristics of the study cohort.

	Patient sample for primary objective		Patient sample for secondary objective	
	N = 4,595		N = 1,740	
1-year pre-index LLT use (n, %)*	N	%	N	%
**Any LLT**	4,046	88.05%	1,531	87.99%
PCSK9i	2	0.04%	1	0.06%
Statin only	3,934	85.61%	1,476	84.83%
High-intensity statin	3,163	68.84%	1,180	67.82%
Moderate- intensity statin	761	16.56%	291	16.72%
Low-intensity statin	10	0.22%	5	0.29%
Statin+Ezetimibe	108	2.35%	55	3.16%
High-intensity statin	80	1.74%	40	2.30%
Moderate-intensity statin	28	0.61%	15	0.86%
Low-intensity statin	0	0.00%	0	0.00%
Ezetimibe only	4	0.09%	0	0.00%
No LLT	549	11.95%	209	12.01%
**Charlson Comorbidity Index Score: (n, %)**				
0	114	2.48%	49	2.82%
1	2,518	54.80%	967	55.57%
2	1,500	32.64%	594	34.14%
3+	463	10.08%	130	7.47%
Mean (SD)	1.6 (1.0)		1.5 (0.8)	
Median	1.0		1.0	
**ASCVD Diagnosis**^†^ **(n, %)**				
Acute Myocardial Infraction (MI)	255	5.55%	101	5.80%
Unstable angina (UA) Hospitalization	398	8.66%	136	7.82%
Stable angina Hospitalization	411	8.94%	150	8.62%
Ischemic stroke (IS)	78	1.70%	23	1.32%
Transient ischemic attack (TIA)	26	0.57%	9	0.52%
Coronary revascularization (PCI)	985	21.44%	341	19.60%
Coronary revascularization (CABG)	141	3.07%	52	2.99%
Coronary revascularization (Other)	3	0.07%	2	0.11%
PAD	64	1.39%	24	1.38%
Symptomatic PAD	5	0.11%	2	0.11%
Non-symptomatic PAD	61	1.33%	23	1.32%
Other ASCVD	1,682	36.61%	634	36.44%
ASCVD other than "other ASCVD"	1,075	23.39%	364	20.92%
Any ASCVD	2,757	60.00%	998	57.36%
Acute coronary syndrome (MI or UA)	616	13.41%	223	12.82%
Stroke (IS or TIA)	95	2.07%	28	1.61%
Revascularization (PCI or CABG or Others)	1,089	23.70%	382	21.95%
**ACC Comorbidities of interest**^†^ **(n, %)**				
At least one comorbidity	3,420	74.43%	1,317	75.69%
Type 2 diabetes *mellitus*	2,055	44.72%	796	45.75%
Hypertension	2,959	64.40%	1,139	65.46%
CKD stage 1–5, unspecified	191	4.16%	52	2.99%
Heart failure	346	7.53%	120	6.90%
Hemodialysis	34	0.74%	8	0.46%
**Recent MACE events**^†^ **(n, %)**				
At least one MACE event	577	12.56%	209	12.01%
Myocardial Infarction	5	0.11%	3	0.17%
Unstable angina	130	2.83%	48	2.76%
Revascularization—PCI	379	8.25%	126	7.24%
Revascularization—CABG	63	1.37%	29	1.67%
Revascularization—Other	3	0.07%	2	0.11%
Ischemic Stroke	11	0.24%	5	0.29%
**Pre-index 6-month LDL-C (mg/dL)**				
*Number of Unique patients with Pre-Index LDL-C value from secondary sample*			1,404	80.69%
Mean (SD)			142.6 (61.4)	
Median			135.0	
LDL-C <70 mg/dL			101	7.19%
LDL-C 70 to <100 mg/dL			206	14.67%
LDL-C 100 to <130 mg/dL			333	23.72%
LDL-C 130 to <160 mg/dL			341	24.29%
LDL-C 160 to <190 mg/dL			210	14.96%
LDL-C > = 190 mg/dL			213	15.17%

ACC—American College of Cardiology; AMI- Acute Myocardial Infraction; ASCVD—Atherosclerotic Cardiovascular Disease; CABG—Coronary artery bypass grafting; CKD–Chronic Kidney Disease; IS—Ischemic stroke; LDL-C—Low-density Lipoprotein Cholesterol; LLT—Lipid Lowering Therapy; MACE—Major adverse cardiovascular events; PAD—Peripheral Artery Disease; PCI—Percutaneous Coronary Intervention; PCSK9i- Protein Convertase Subtilisin/kexin Type 9 Inhibitors; TIA—Transient Ischemic Attack; UA—Unstable angina.

*PCSK9i/Statin/Eze use and intensity were measured using a 1-year pre-index look back period.

^†^ASCVD diagnoses were measured during the 1-year pre-index period using Dubai Real-World Claims. Unstable angina is identified through IP claims only; Other ASCVD diagnoses are identified by at least one confirmatory (i.e., non-ancillary) medical claim with ICD-9/ICD-10 diagnosis codes for ASCVD conditions. Comorbidities of interest were measured during the 1-year pre-index period using Dubai Real-World Claims. MACE events were measured during the 1-year pre-index period using Dubai Real-World Claims (Primary diagnoses from IP claims and any non-ancillary diagnoses from OP claims).

In the first year after index MI, 387 (8.42%) patients experienced at least one recurrent MI, 135 (2.94%) patients had at least one revascularization, 122 (2.66%) patients had at least one UA hospitalization and 9 (0.20%) patients experienced at least one IS (Table 3). The rate of individual MACE per 100 person-years was higher for UA (5.16 new events/100 PYs; 95% CI 4.25–6.08 cases/100 PYs); followed by recurrent MI (3.26 new events/100 person-years [PYs]; 95% CI 2.53–3.99 cases/100 PYs); revascularization (0.55 new events/100 PYs; 95% CI 0.25–0.85 cases/100 PYs) and IS (0.38 new events/100 PYs; 95% CI 0.13–0.63 cases/100 PYs). Rate of composite MI/IS events was 3.64 new events/100 PYs (95% CI 2.87–4.41 cases/100 PYs) and the rate of composite of any MACE (including MI/IS/Revascularization/UA hospitalization) was 6.77 new events/100 PYs; (95% CI 5.72–7.82 cases/100 PYs) (Table 3).

**Table 3:** One-year MACE rate among all patients discharged with MI.

CV Events*	Patients with at least 1 MACE		Number distinct MACE^†^	Patient-years^‡^	MACE rate	Rate per 100 patient-years	95% CI of rate per 100 patient-years	
	N	%					Lower Limit	Upper Limit
MI	387	8.42	77	2,363.1	0.0326	3.26	2.53	3.99
IS	9	0.20	9	2,363.1	0.0038	0.38	0.13	0.63
UA Hospitalization	122	2.66	122	2,363.1	0.0516	5.16	4.25	6.08
Revascularization	135	2.94	13	2,363.1	0.0055	0.55	0.25	0.85
Composite MI/IS rate	394	8.57	86	2,363.1	0.0364	3.64	2.87	4.41
Composite MACE rate	589	12.82	160	2,363.1	0.0677	6.77	5.72	7.82

IS–Ischemic Stroke; MACE–Major Adverse Cardiovascular Event; MI–Myocardial Infraction; UA–Unstable Angina.

*MI is assessed using Inpatient (IP) events (Diagnosis at primary position only); IS is assessed using IP events (Diagnosis at primary position only); UA Hospitalizations are assessed using IP events (Diagnosis at primary position only); Revascularizations are assessed using IP/Outpatient (OP) events (Diagnosis at any position); Composite of MI or IS events is assessed using MI/IS events (IP only, diagnosis at primary position); Composite all MACE rate—MI, UA, Revasc occurring within 30 days of each other were considered as same event where as events occurring outside 30 days is considered as distinct events. IS occurring within 30 days of MI, UA or Revasc were considered as distinct event since it is cerebrovascular in nature.

^†^Total no of distinct MACE—after observation of the first CV event, all subsequent CV events of the same type (MI (IP only) after a previous MI (IP only), IS (IP only) after a previous IS (IP only), UA (IP only) after a previous UA (IP only)) are counted as the same episode as long as they are within 30 days of the discharge date of previous event. Revascularization (IP or OP) occurring within 30 days of discharge from prior MI/IS/UA Hosp or prior revascularization will not be considered as a distinct event; ^‡^Total patient-years are calculated as: Time from index date until the first occurrence of: a) End of reporting period (1-year post-index period) b) End of study period (Dec 31, 2019) or c) End of Continuous Eligibility (CE).

In the 6-months after index MI, patients presented on average with LDL-C levels of 88.3 mg/dL (SD 43.5 mg/dL), with the majority presenting with LDL-C levels ≥ 70 mg/dL (N = 1,051/1,740; 60.40%) (Table 4).

**Table 4:** Post-index LDL-C levels of patients discharged with MI.

	Post-index LDL-C	
	(Last value in 6-month post-MI)	
** LDL-C (mg/dL)** ^*^	**N = 1,740**	
Mean (SD)	88.3 (43.5)	
Median	78.5	
	**N**	**%**
LDL-C <70 mg/dL	689	39.60%
LDL-C 70 to <100 mg/dL	526	30.23%
LDL-C 100 to <130 mg/dL	278	15.98%
LDL-C 130 to <160 mg/dL	137	7.87%
LDL-C 160 to <190 mg/dL	63	3.62%
LDL-C > = 190 mg/dL	47	2.70%

LDL-C—Low-density Lipoprotein Cholesterol.

*Last ever LDL-C in 6-months post-index period; LDL-C—Low-density Lipoprotein Cholesterol.

In the 12-months after index MI, 93.45% of the patients (N = 4,294) received LLT; mainly statins (N = 4,082; 88.84%) and those of high-intensity (N = 3,115; 67.79%). A minority of patients received a combination of statin + ezetimibe (N = 209; 4.55%), 9 (0.20%) patients received PCSK9i and 3 (0.07%) patients received ezetimibe alone (Table 5). The LLT prescription patterns observed in the 1-, 3- and 6-months post-index MI were similar (S3 Table).

**Table 5:** One-year post-index LLT patterns among all patients discharged with MI.

Post-index Treatment Characteristics	All patients in sample for primary objective	
	N = 4,595	
	N	%
12- Month Post-index LLT use^*^ **(n, %)**	4,595	100.00%
Any LLT	4,294	93.45%
PCSK9i	9	0.20%
Statin only	4,082	88.84%
High-intensity statin	3,115	67.79%
Moderate-intensity statin	963	20.96%
Low-intensity statin	4	0.09%
Statin+Ezetimibe	209	4.55%
High-intensity statin	169	3.68%
Moderate-intensity statin	39	0.85%
Low-intensity statin	1	0.02%
Ezetimibe only	3	0.07%

CE–Continuous Eligibility; LLT—Lipid Lowering Therapy; PCSK9i - Protein Convertase Subtilisin/kexin type 9 Inhibitors.

Note: Index date is included in the post-index period. First prescription in 1-month post-index period and last prescription in 12-month post-index period were used.

^*^Patients who had at least 1 prescription for LLT during the 12-month post-index period; Denominator was number of patients in sample for primary objective with at least 1 claim in 12 months CE in post-index period.

Table 6 presents the changes in LLT use in the 12-months post-index MI, comparing to the 3-months before MI. The majority of patients continued on the statin therapy they were on prior to the index MI event (N = 2,745; 59.74%); with a minority of patients initiating LLT (N = 474; 10.32%), discontinuing it (N = 182; 3.96%) or changing statin intensity (either increasing it [10.77%] or reducing it [10.03%]). A minority of patients augmented their statin therapy with ezetimibe (N = 106; 2.31%) and 5 patients switched treatment to a PCSK9i (Table 6). The changes in LLT use were similar in the 1- and 6-months post-index MI, comparing to the 3-months before MI (S4 Table).

**Table 6:** Post-index changes in LLT patterns among all patients discharged with MI.

	All patients in sample for primary objective	
	N = 4,595	
Post-index LLT Changes	N	%
**Changes in LLT use from first Rx during 3-month pre-index period to last Rx in 12-mon post-index (n, %)**		
Patients with 12-month post-index CE^*^	4,595	100.00%
LLT initiation	474	10.32%
No LLT initiation	119	2.59%
Discontinuation	182	3.96%
Statin Intensified	495	10.77%
Statin Lowered	461	10.03%
Statin added to Eze	106	2.31%
Eze added to Statin	2	0.04%
Statin switch to Eze	1	0.02%
Eze switch to Statin	1	0.02%
Statin (no-change)	2,745	59.74%
Eze (no-change)	-	0.00%
Statin discontinuation	1	0.02%
Switch to PCSK9i	5	0.11%
PCSK9i (no-change)	2	0.04%
PCSK9i Intensified	1	0.02%

EZE- Ezetimibe; LLT—Lipid Lowering Therapy; PCSK9i - Protein Convertase Subtilisin/kexin type 9 inhibitors; Rx—Prescription.

*Patients with 12-month post-index CE. Patients with at least one claim for any service (i.e., Drug, Procedures, consultation etc. in any market CVD or non-CVD) in 12-month post-index period, these patients were selected and the change in LLT use is reported.

## Discussion

This retrospective cohort study included 4,595 adult patients from the Dubai Real-World Claims Database, discharged with MI and followed-up to their first-year post-index MI. In the 12 months before MI discharge, most patients presented with an ASCVD diagnosis along with comorbid cardiovascular risk factors such as hypertension, diabetes and hypercholesterolemia; which may justify the high rates of baseline LLT use, mostly high-intensity statin therapy (Table 2). These results are in line with the evidence from large-scale randomized trials showing that statin therapy reduces the risk of MACE by about one-quarter for each mmol/L reduction in LDL-C during each year (after the first) that it continues to be taken, in both ASCVD primary and secondary prevention [14].

In the first year after MI discharge, 8.42% of the patients experienced at least one recurrent MI and 12.82% experienced at least one MACE (composite rate 6.77 events/100 PYs) (Table 3). Other studies have found that around 20% of patients with MI experience at least one MACE in the first year after index MI; approximately 12% of these patients having at least one recurrent MI [15, 16]. Nonetheless, more contemporary data on MACE rates shows lower incidence rates of MACE, even in patients with established ASCVD (2.12 events/100 PYs) [17]. Therefore, the results of this study indicate that patients hospitalized with MI in Dubai present with an increased risk of MACE in their first year post-discharge, than that reported in international studies, suggesting a need for improving patient health outcomes in this region. Comparison of LDL-C levels in the pre-index and post-index periods shows reduction in LDL-C levels. We see that the % of patients in the range of better LDL-C control from <70 mg/dl to <100 mg/dl has improved drastically from 21.86% in the pre-index to 69% to post-index period. Likewise, the % of patients in the fairly poor control >100mg/dl to <190 mg/dl also reduced from 62.97% in the pre-index to 27.4% in the post-index period. The % of patients with very poor control >190 mg/dl reduced drastically from 15.17% to 2.7% from the pre-index to post-index period. This demonstrates that post the event of MI the patients have demonstrated fairly better control along with the demonstrated intensification of LLT shown in Tables 5 and 6.

However, if we take into consideration the goal of achieving LDL-C levels <70mg/dL to achieve good clinical outcomes, the percentage of patients who achieved <70mg/dL was not substantial (pre-index period: 7.19%; post-index period; 39.60%) (Tables 3 and 4). After their index MI, the majority (60.40%) of patients presented with LDL-C levels ≥ 70 mg/dL (Table 4); despite most of them receiving high-intensity statin therapy, with only a minority of patients receiving a combination of ezetimibe with the statin therapy or a PCSK9 inhibitor (Tables 5 and 6).

In light of the increased rate of MACE observed in the first year after MI and given that the majority of patients presented with LDL-C levels ≥70 mg/dL despite most of them being on high-intensity statin therapy; our study findings underscore potential opportunities to improve clinical outcomes for patients with MI, by continued improvement in cardiac rehabilitation and optimization of LLT prescription, particularly in high-risk patients. Current guidelines emphasize on considering addition of non-statins including ezetimibe or a PCSK9 inhibitor to maximally tolerated statin therapy, in very high-risk ASCVD patients with LDL-C levels of ≥ 70mg/dL [18]. Numerous real-world studies report failure to meet guideline recommended LDL-C levels with statin therapy alone, particularly for high-risk patients [19–23]. However, in line with our results, other drug utilization studies have reported that fewer than 1% of patients with ASCVD and/or heterozygous familial hypercholesterolemia added ezetimibe to statin therapy, and fewer than 1% of patients were prescribed PCSK9 inhibitors [24]. Many factors may contribute to the low rates of non-statin LLT, and the reasons for LLT initiation and changes were not investigated in this study. Nonetheless, the results from our study indicate a high-risk of CVEs during the first year post-MI discharge. These results highlight the need for better strategies to improve lipid control such as addition of non-statin therapies.

The results of this study should be interpreted in the context of its limitations. Important sources of bias should be considered in the interpretation of the results of this study: 1) Since medical conditions were identified based on existing records, coding inaccuracies may lead to misclassification bias; 2) Misclassification of LLT use is also possible since low-intensity statins may be available over the counter. However, it is likely that most prescriptions are issued in clinical care and recorded, especially among patients covered by private health insurance. In addition, it should be noted that no data were available on potential non-adherence to LLT and other lifestyle measures to improve cholesterol levels, such as a balanced diet and physical activity. As such, the effect of those factors on the observed LDL-C levels and LLT patterns is unknown. Moreover, only 37.87% (N = 1,740) of the patients had at least one LDL-C measurement in the 6-month after MI. This could represent a more intense surveillance or screening for a group of high-risk patients, and therefore introduce ascertainment bias in the interpretation of the study findings [25].

Despite these limitations, this study provides valuable evidence on the patterns of LDL-C and LLT use in the first year after MI, and the risk of subsequent MACE in survivors of MI in the Middle East. The evidence highlights the need for more aggressive treatment approaches such as augmenting statin therapy with ezetimibe or initiating PCSK9 inhibitors to achieve additional LDL-C reduction and reduce risk of recurrent MACE. The findings of this study highlight the potential to improve the clinical management of patients with MI in UAE, providing valuable evidence to inform primary and secondary prevention of ASCVD in this region. Future studies should identify barriers to suboptimal prescription of non-statin LLT and provide guidance to improve it.

## Supporting information

S1 File (DOCX)

S1 Appendix Expanded methods. (DOCX)

S2 Appendix Data transformation. (DOCX)

S3 Appendix Handling of missing data. (DOCX)

S1 Table Statin intensity classification. (DOCX)

S2 Table Treatment characteristics—90-day pre-index LLT use. (DOCX)

S3 Table Post-index LLT treatment patterns among all patients discharged with MI. (DOCX)

S4 Table Post-index changes in LLT patterns among all patients discharged with MI. (DOCX)
