# Supplementary material for: Lipid lowering therapy patterns and the risk of cardiovascular events in the 1-year after acute myocardial infarction in United Arab Emirates
Source: PLoS One. 2022 Sep 2;17(9):e0268709. doi: 10.1371/journal.pone.0268709 (PMC9439245; doi:10.1371/journal.pone.0268709)
Supplement: S1 File — (DOCX) [file pone.0268709.s001.docx]

**Supplementary Materials**

**A) Expanded Methods**

**1. Code Lists**

In the Dubai Real World Claims Database, the medical claims are coded using International Classification of Diseases – Tenth Revision – Clinical Modification (ICD 10-CM), Current Procedural Terminology (CPT 4), and the Dubai Drug Coding System for Pharmacy claims.

The following code lists were used in this study:

- CKD stage 1-5, unspecified: ICD10 diagnosis codes: N18.1, N18.2, N18.3, N18.4, N18.5, N18.9 (any diagnosis)
- Heart failure: ICD10 diagnosis codes: I50.x, I50.xx (any diagnosis)
- Hemodialysis: Maintenance and peritoneal dialysis were identified from Dubai Real World Claims.ICD10 diagnosis codes: Z49.x, Z49.xx, Z99.2, Z91.15; (relevant CPT and ICD10 procedure codes not available)
- Stable Angina: I20.1, I20.8, I20.9, I23.7, I25.111, I25.118, I25.119, I25.701, I25.708, I25.709, I25.711, I25.718, I25.719, I25.721, I25.728, I25.729, I25.731, I25.738, I25.739, I25.751 , I25.758, I25.759, I25.761, I25.768, I25.769, I25.791, I25.798, I25.799
- Unstable Angina: I20.0, I24.0, I24.8, I25.110, I25.700, I25.710, I25.720, I25.730, I25.750, I25.760, I25.790
- Other ASCVD: I25.6, I25.10, I25.5, I25.810, I25.811, I25.812, I25.82, I25.83, I25.89, I25.84, I25.9
- Asymptomatic PAD: Z95.820, Z98.62, Z95.828, I70.1, I70.29x, I70.40x, I70.49x, I70.50x, I70.59x, I70.92 , I70.8, I70.90, I70.91, I75.01x, I75.02x, I75.8x, I70.0, I70.20x, I70.30x, I70.39x, I70.60x, I70.69x, I70.70x, I70.79x, I73.9, I73.1, I73.89
- Symptomatic PAD: I70.21x, I70.22x, I70.23x, I70.24x, I70.25, I70.26x, I70.41x, I70.42x, I70.43x, I70.44x, I70.45, I70.46x, I70.511, I70.512, I70.513, I70.518, I70.519, I70.52x, I70.53x, I70.54x, I70.55, I70.56x, I70.31x, I70.32x, I70.33x, I70.34x, I70.35, I70.36x, I70.61x, I70.62x, I70.63x, I70.64x, I70.65, I70.66x, I70.71x, I70.72x, I70.73x, I70.74x, I70.75, I70.76x, I71.1, I71.3, I71.5, I71.8
- Coronary revascularization CABG: Z95.1 (CPT Codes: 33510, 33511, 33512, 33513, 33514, 33516, 33517, 33518, 33519, 33521, 33522, 33523, 33533, 33534, 33535, 33536, 33508, S2205, S2206, S2207, S2208, S2209)
- Familial Hypercholesterolemia: E78.0
- Hyperlipidemia: E78.0, E78.2, E78.4, E78.5 (E78.00 not available)
- Coronary revascularization PCI: Z98.61, Z95.5 (CPT Codes: 92973, C9600, C9601, 92920, 92921, 92928, 92929, 92924, 92925, 92933, 92934, 92937, 92938, 92941, 92943, 92944, C9602, C9603, C9604, C9605, C9606, C9607, C9608, 92975, 92977)
- Coronary revascularization Others: 33140, 33141, 33572
- TIA: G45.x, G46.0, G46.1, H34.00, H34.01, H34.02, H34.03, I67.841, I67.848
- Myocardial infarction: I21.01, I21.02, I21.09, I21.11, I21.19, I21.21, I21.29, I21.3, I21.4, I22.0, I22.1, I22.2, I22.8, I22.9, I25.2
- IS: I63.00 , I63.x , I63.02 , I63.03x , I63.09 , I63.20 , I63.21x, I63.22 , I63.23x , I63.29 , I63.30 , I63.31x, I63.32x, I63.33x, I63.34x, I63.39 , I63.50 , I63.51x, I63.52x, I63.53x, I63.54x, I63.59 , I63.6 , I63.8 , I63.9 , Z86.73
- T2DM: E11, E11.0, E11.00, E11.01, E11.2, E11.21, E11.22, E11.29, E11.3, E11.31, E11.311, E11.319, E11.32, E11.321, E11.329, E11.33, E11.331, E11.339, E11.34, E11.341 , E11.349, E11.35, E11.351, E11.352, E11.353, E11.354, E11.355, E11.359, E11.36, E11.37, E11.37X, E11.39, E11.4, E11.40, E11.41, E11.42, E11.43, E11.44, E11.49, E11.5 , E11.51, E11.52, E11.59, E11.6, E11.61, E11.610, E11.618, E11.62, E11.620, E11.621, E11.622, E11.628, E11.63, E11.630, E11.638, E11.64, E11.641, E11.649, E11.65, E11.69, E11.8, E11.9
- Hypertension: I10, I11, I11.9, I12, I12.0, I12.9, I13, I13.1, I13.10, I13.11, I15, I15.0, I15.1, I15.2, I15.8, I15.9

## 2. Data transformation

The following definitions and data transformation/calculation were used in this study:

### Cardiovascular Events (CVEs)

- **Events in the 1-year period post-MI:** The total number of acute CVEs in the 1-year period after MI, including the events occurring on the index date was assessed. The following types of post-MI acute CVEs were considered:
  - MI (IP only, primary position)
  - IS (IP only, primary position)
  - Revascularization (IP or OP, any position)
  - UA Hospitalization (IP only, primary position)
  - Composite of MI (IP only, primary position) or IS (IP only, primary position)
- **Multiple CVEs**: In this study, a CVE must have occurred at least 30 days after the previous acute CVE of the same type to be counted as a distinct CVE, considering the following:
- After observation of the first acute CVE, all subsequent acute CVE of the same type (MI (IP only) after a previous MI (IP only), IS (IP only) after a previous IS (IP only), UA (IP only) after a previous UA (IP only)) were counted as the same episode as long as they were within 30 days of the discharge date of previous event.
  - Revascularization (IP or OP) occurring within 30 days of discharge from prior MI/IS/UA Hospital or prior revascularization was not considered as a distinct event.

### 3. Statin Intensity

Table SI presents the statin intensity classification adopted in this study.

Table S1: Statin intensity classification

| **Statin therapy** | **Brand Strength** | | | |
| --- | --- | --- | --- | --- |
|  | **Low**  **Intensity** | **Moderate**  **Intensity** | **High**  **Intensity** | **Notes**  **(classification of atypical doses)** |
| Atorvastatin | <10 mg | 10 to <40 mg | ≥40 mg | 30 mg = Moderate intensity |
| Fluvastatin | <80 mg | 80 mg | n/a | 10 mg = Low intensity |
| Lovastatin | <40 mg | ≥40 mg | n/a | 10 mg = Low intensity 80 mg = Moderate intensity |
| Pitavastatin | <2 mg | ≥2 mg | n/a | - |
| Pravastatin | <40 mg | ≥40 mg | n/a | <10 mg = Low intensity |
| Rosuvastatin | <5 mg | 5 to <20 mg | ≥20 mg | <5 mg = Low intensity 15 mg = Moderate intensity |
| Simvastatin | <20 mg | 20 to <80 mg | ≥80 mg | <20 mg = Low intensity >40 to <80 mg = Moderate ≥80 mg = High intensity |

n/a – not applicable

### 4. Handling of missing data

A clinical and statistical algorithm was used to fill the missing patient demographics (age and gender). Firstly, age was imputed based on a clinical algorithm and for the remaining patients a statistical algorithm was used. With regards to the missing data on therapy duration, the following steps were followed:

1. All the claims having therapy duration were extracted
2. The frequency of claims by therapy duration for one pack by each product and form separately were calculated
3. All the missing therapy duration using the highest frequency therapy duration selected in the above step was replaced.

**B) Supplementary Tables**

Table S2: Treatment characteristics - 90-day pre-index LLT use

|  | **Patients’ sample for primary objective** | | **Patients’ sample for secondary objective** | |
| --- | --- | --- | --- | --- |
|  | **N=4,595** | | **N=1,740** | |
| **90-day pre-index LLT use (n, %)^*^** |  |  |  |  |
| **Any LLT** | 4,002 | 87.09% | 1,514 | 87.01% |
| PCSK9i | 2 | 0.04% | 1 | 0.06% |
| Statin only | 3,908 | 85.05% | 1,466 | 84.25% |
| High statin intensity | 3,163 | 68.84% | 1,178 | 67.70% |
| Medium statin intensity | 736 | 16.02% | 283 | 16.26% |
| Low statin intensity | 9 | 0.20% | 5 | 0.29% |
| Statin+Ezetimibe | 90 | 1.96% | 48 | 2.76% |
| High statin intensity | 69 | 1.50% | 35 | 2.01% |
| Medium statin intensity | 21 | 0.46% | 13 | 0.75% |
| Low statin intensity | 0 | 0.00% | 0 | 0.00% |
| Ezetimibe only | 4 | 0.09% | 0 | 0.00% |
| No LLT | 593 | 12.91% | 226 | 12.99% |

LLT - Lipid Lowering Therapy; PCSK9i- Protein convertase subtilisin/kexin type 9 inhibitors;

^*^PCSK9i/Statin/Eze use and intensity were measured using a 90-day pre-index look back period

Table S3. Post-index LLT treatment patterns among all patients discharged with MI

| **Post-index Treatment Characteristics** | **All patients in sample for primary objective** | |
| --- | --- | --- |
|  | **N=4,595** | |
|  | **N** | **%** |
| **3- Month Post-index LLT use^*^ (n, %)** |  |  |
| **Patients with 3-month post index CE** | 4,539 | 100.00% |
| Any LLT | 4,164 | 91.74% |
| PCSK9i | 4 | 0.09% |
| Statin only | 4,031 | 88.81% |
| High statin intensity | 3,297 | 72.64% |
| Medium statin intensity | 730 | 16.08% |
| Low statin intensity | 4 | 0.09% |
| Statin+Ezetimibe | 129 | 2.84% |
| High statin intensity | 108 | 2.38% |
| Medium statin intensity | 21 | 0.46% |
| Low statin intensity | - | 0.00% |
| Ezetimibe only | 4 | 0.09% |
| **6- Month Post-index LLT use^†^ (n, %)** |  |  |
| **Patients with 6-month post index CE** | 4,595 | 100.00% |
| Any LLT | 4,270 | 92.93% |
| PCSK9i | 5 | 0.11% |
| Statin only | 4,100 | 89.23% |
| High statin intensity | 3,233 | 70.36% |
| Medium statin intensity | 862 | 18.76% |
| Low statin intensity | 5 | 0.11% |
| Statin+Ezetimibe | 167 | 3.63% |
| High statin intensity | 137 | 2.98% |
| Medium statin intensity | 29 | 0.63% |
| Low statin intensity | 1 | 0.02% |
| Ezetimibe only | 3 | 0.07% |

CE – Continuous Eligibility; LLT - Lipid Lowering Therapy; PCSK9i - Protein convertase subtilisin/kexin type 9 inhibitors

Note: Index date is included in the post-index period. First prescription in 1- month post-index period and last prescription in 3-month, 6 month or 12 month post-index period were used.

^*^Patients who had at least 1 prescription for LLT during the 3-month post-index period; Denominator was number of patients in sample for primary objective with at least 1 claim during 3-month CE in post-index period

**^†^**Patients who had at least 1 prescription for LLT during the 6-month post-index period; Denominator was number of patients in sample for primary objective with at least 1 claim during 6-month CE in post-index period

Table S4. Post-index changes in LLT patterns among all patients discharged with MI

|  | **All patients in sample**  **for primary objective**  **N=4,595** | |
| --- | --- | --- |
| **Post-index LLT Changes** | **N** | **%** |
| **Changes in LLT use from first Rx during 3-month pre-index period to last Rx in 1-mon post-index (n, %)** | | |
| **Patients with 1-month post index CE^*^** | 4,425 | 100.00% |
| LLT initiation | 410 | 9.27% |
| No LLT initiation | 152 | 3.44% |
| Discontinuation | 311 | 7.03% |
| Statin Intensified | 405 | 9.15% |
| Statin Lowered | 161 | 3.64% |
| Statin to Eze Augmentation | 16 | 0.36% |
| Eze to Statin Augmentation | 1 | 0.02% |
| Statin switch to Eze | 1 | 0.02% |
| Eze switch to Statin | 1 | 0.02% |
| Statin Same | 2,961 | 66.92% |
| Eze Same | 1 | 0.02% |
| Statin drop off | 1 | 0.02% |
| Switch to PCSK9i | 1 | 0.02% |
| PCSK9i Same | 2 | 0.05% |
| PCSK9i Intensified | 1 | 0.02% |
| **Changes in LLT use from first Rx during 1-mon post-index period to last Rx in 6-mon post-index (n, %)** | | |
| **Patients with 6-month post index CE^b^** | 4,595 | 100.00% |
| LLT initiation | 184 | 4.00% |
| No LLT initiation | 325 | 7.07% |
| Discontinuation | - | 0.00% |
| Statin Intensified | 365 | 7.94% |
| Statin Lowered | 399 | 8.68% |
| Statin to Eze Augmentation | 62 | 1.35% |
| Eze to Statin Augmentation | - | 0.00% |
| Statin switch to Eze | - | 0.00% |
| Eze switch to Statin | 1 | 0.02% |
| Statin Same | 3,252 | 70.77% |
| Eze Same | 2 | 0.04% |
| Statin drop off | - | 0.00% |
| Switch to PCSK9i | 1 | 0.02% |
| PCSK9i Same | 4 | 0.09% |
| PCSK9i Intensified | - | 0.00% |
| **Changes in LLT use from first Rx during 1-mon post-index period to last Rx in 12-mon post-index (n, %)** | | |
| **Patients with 12-month post index CE^c^** | 4,595 | 100.00% |
| LLT initiation | 208 | 4.53% |
| No LLT initiation | 301 | 6.55% |
| Discontinuation | - | 0.00% |
| Statin Intensified | 356 | 7.75% |
| Statin Lowered | 485 | 10.55% |
| Statin to Eze Augmentation | 99 | 2.15% |
| Eze to Statin Augmentation | 1 | 0.02% |
| Statin switch to Eze | - | 0.00% |
| Eze switch to Statin | - | 0.00% |
| Statin Same | 3,135 | 68.23% |
| Eze Same | 2 | 0.04% |
| Statin drop off | - | 0.00% |
| Switch to PCSK9i | 4 | 0.09% |
| PCSK9i Same | 4 | 0.09% |
| PCSK9i Intensified | - | 100.00% |

EZE- ezetimibe; LLT - lipid Lowering Therapy; PCSK9i - Protein convertase subtilisin/kexin type 9 inhibitors; Rx - prescription

^*^Patients with 1-month post index CE. Patients with at least one claim for any service (i.e., Drug, Procedures, consultation etc. in any market CVD or non-CVD) in 1-month post index period, these patients were selected and the change in LLT use is reported.
