# Supplementary material for: Lipid lowering therapy patterns and the risk of cardiovascular events in the 1-year after acute myocardial infarction in United Arab Emirates
Source: PLoS One. 2022 Sep 2;17(9):e0268709. doi: 10.1371/journal.pone.0268709 (PMC9439245; doi:10.1371/journal.pone.0268709)
Supplement: S1 Appendix — (DOCX) [file pone.0268709.s002.docx]

**S1 Appendix: Expanded Methods**

**1. Code Lists**

In the Dubai Real World Claims Database, the medical claims are coded using International Classification of Diseases – Tenth Revision – Clinical Modification (ICD 10-CM), Current Procedural Terminology (CPT 4), and the Dubai Drug Coding System for Pharmacy claims.

The following code lists were used in this study:

- CKD stage 1-5, unspecified: ICD10 diagnosis codes: N18.1, N18.2, N18.3, N18.4, N18.5, N18.9 (any diagnosis)
- Heart failure: ICD10 diagnosis codes: I50.x, I50.xx (any diagnosis)
- Hemodialysis: Maintenance and peritoneal dialysis were identified from Dubai Real World Claims.ICD10 diagnosis codes: Z49.x, Z49.xx, Z99.2, Z91.15; (relevant CPT and ICD10 procedure codes not available)
- Stable Angina: I20.1, I20.8, I20.9, I23.7, I25.111, I25.118, I25.119, I25.701, I25.708, I25.709, I25.711, I25.718, I25.719, I25.721, I25.728, I25.729, I25.731, I25.738, I25.739, I25.751 , I25.758, I25.759, I25.761, I25.768, I25.769, I25.791, I25.798, I25.799
- Unstable Angina: I20.0, I24.0, I24.8, I25.110, I25.700, I25.710, I25.720, I25.730, I25.750, I25.760, I25.790
- Other ASCVD: I25.6, I25.10, I25.5, I25.810, I25.811, I25.812, I25.82, I25.83, I25.89, I25.84, I25.9
- Asymptomatic PAD: Z95.820, Z98.62, Z95.828, I70.1, I70.29x, I70.40x, I70.49x, I70.50x, I70.59x, I70.92 , I70.8, I70.90, I70.91, I75.01x, I75.02x, I75.8x, I70.0, I70.20x, I70.30x, I70.39x, I70.60x, I70.69x, I70.70x, I70.79x, I73.9, I73.1, I73.89
- Symptomatic PAD: I70.21x, I70.22x, I70.23x, I70.24x, I70.25, I70.26x, I70.41x, I70.42x, I70.43x, I70.44x, I70.45, I70.46x, I70.511, I70.512, I70.513, I70.518, I70.519, I70.52x, I70.53x, I70.54x, I70.55, I70.56x, I70.31x, I70.32x, I70.33x, I70.34x, I70.35, I70.36x, I70.61x, I70.62x, I70.63x, I70.64x, I70.65, I70.66x, I70.71x, I70.72x, I70.73x, I70.74x, I70.75, I70.76x, I71.1, I71.3, I71.5, I71.8
- Coronary revascularization CABG: Z95.1 (CPT Codes: 33510, 33511, 33512, 33513, 33514, 33516, 33517, 33518, 33519, 33521, 33522, 33523, 33533, 33534, 33535, 33536, 33508, S2205, S2206, S2207, S2208, S2209)
- Familial Hypercholesterolemia: E78.0
- Hyperlipidemia: E78.0, E78.2, E78.4, E78.5 (E78.00 not available)
- Coronary revascularization PCI: Z98.61, Z95.5 (CPT Codes: 92973, C9600, C9601, 92920, 92921, 92928, 92929, 92924, 92925, 92933, 92934, 92937, 92938, 92941, 92943, 92944, C9602, C9603, C9604, C9605, C9606, C9607, C9608, 92975, 92977)
- Coronary revascularization Others: 33140, 33141, 33572
- TIA: G45.x, G46.0, G46.1, H34.00, H34.01, H34.02, H34.03, I67.841, I67.848
- Myocardial infarction: I21.01, I21.02, I21.09, I21.11, I21.19, I21.21, I21.29, I21.3, I21.4, I22.0, I22.1, I22.2, I22.8, I22.9, I25.2
- IS: I63.00 , I63.x , I63.02 , I63.03x , I63.09 , I63.20 , I63.21x, I63.22 , I63.23x , I63.29 , I63.30 , I63.31x, I63.32x, I63.33x, I63.34x, I63.39 , I63.50 , I63.51x, I63.52x, I63.53x, I63.54x, I63.59 , I63.6 , I63.8 , I63.9 , Z86.73
- T2DM: E11, E11.0, E11.00, E11.01, E11.2, E11.21, E11.22, E11.29, E11.3, E11.31, E11.311, E11.319, E11.32, E11.321, E11.329, E11.33, E11.331, E11.339, E11.34, E11.341 , E11.349, E11.35, E11.351, E11.352, E11.353, E11.354, E11.355, E11.359, E11.36, E11.37, E11.37X, E11.39, E11.4, E11.40, E11.41, E11.42, E11.43, E11.44, E11.49, E11.5 , E11.51, E11.52, E11.59, E11.6, E11.61, E11.610, E11.618, E11.62, E11.620, E11.621, E11.622, E11.628, E11.63, E11.630, E11.638, E11.64, E11.641, E11.649, E11.65, E11.69, E11.8, E11.9
- Hypertension: I10, I11, I11.9, I12, I12.0, I12.9, I13, I13.1, I13.10, I13.11, I15, I15.0, I15.1, I15.2, I15.8, I15.9
