# Supplementary material for: Lipid lowering therapy patterns and the risk of cardiovascular events in the 1-year after acute myocardial infarction in United Arab Emirates
Source: PLoS One. 2022 Sep 2;17(9):e0268709. doi: 10.1371/journal.pone.0268709 (PMC9439245; doi:10.1371/journal.pone.0268709)
Supplement: S2 Appendix — (DOCX) [file pone.0268709.s003.docx]

## S2 Appendix: Data transformation

The following definitions and data transformation/calculation were used in this study:

### Cardiovascular Events (CVEs)

- **Events in the 1-year period post-MI:** The total number of acute CVEs in the 1-year period after MI, including the events occurring on the index date was assessed. The following types of post-MI acute CVEs were considered:
  - MI (IP only, primary position)
  - IS (IP only, primary position)
  - Revascularization (IP or OP, any position)
  - UA Hospitalization (IP only, primary position)
  - Composite of MI (IP only, primary position) or IS (IP only, primary position)
- **Multiple CVEs**: In this study, a CVE must have occurred at least 30 days after the previous acute CVE of the same type to be counted as a distinct CVE, considering the following:
- After observation of the first acute CVE, all subsequent acute CVE of the same type (MI (IP only) after a previous MI (IP only), IS (IP only) after a previous IS (IP only), UA (IP only) after a previous UA (IP only)) were counted as the same episode as long as they were within 30 days of the discharge date of previous event.
  - Revascularization (IP or OP) occurring within 30 days of discharge from prior MI/IS/UA Hospital or prior revascularization was not considered as a distinct event.
