# Supplementary material for: Lipid lowering therapy patterns and the risk of cardiovascular events in the 1-year after acute myocardial infarction in United Arab Emirates
Source: PLoS One. 2022 Sep 2;17(9):e0268709. doi: 10.1371/journal.pone.0268709 (PMC9439245; doi:10.1371/journal.pone.0268709)
Supplement: S3 Appendix — (DOCX) [file pone.0268709.s004.docx]

### S3 Appendix: Handling of missing data

A clinical and statistical algorithm was used to fill the missing patient demographics (age and gender). Firstly, age was imputed based on a clinical algorithm and for the remaining patients a statistical algorithm was used. With regards to the missing data on therapy duration, the following steps were followed:

1. All the claims having therapy duration were extracted
2. The frequency of claims by therapy duration for one pack by each product and form separately were calculated
3. All the missing therapy duration using the highest frequency therapy duration selected in the above step was replaced.
