# Supplementary material for: Lipid lowering therapy patterns and the risk of cardiovascular events in the 1-year after acute myocardial infarction in United Arab Emirates
Source: PLoS One. 2022 Sep 2;17(9):e0268709. doi: 10.1371/journal.pone.0268709 (PMC9439245; doi:10.1371/journal.pone.0268709)
Supplement: S1 Table — (DOCX) [file pone.0268709.s005.docx]

S1 Table: Statin intensity classification

| **Statin therapy** | **Brand Strength** | | | |
| --- | --- | --- | --- | --- |
|  | **Low**  **Intensity** | **Moderate**  **Intensity** | **High**  **Intensity** | **Notes**  **(classification of atypical doses)** |
| Atorvastatin | <10 mg | 10 to <40 mg | ≥40 mg | 30 mg = Moderate intensity |
| Fluvastatin | <80 mg | 80 mg | n/a | 10 mg = Low intensity |
| Lovastatin | <40 mg | ≥40 mg | n/a | 10 mg = Low intensity 80 mg = Moderate intensity |
| Pitavastatin | <2 mg | ≥2 mg | n/a | - |
| Pravastatin | <40 mg | ≥40 mg | n/a | <10 mg = Low intensity |
| Rosuvastatin | <5 mg | 5 to <20 mg | ≥20 mg | <5 mg = Low intensity 15 mg = Moderate intensity |
| Simvastatin | <20 mg | 20 to <80 mg | ≥80 mg | <20 mg = Low intensity >40 to <80 mg = Moderate ≥80 mg = High intensity |

n/a – not applicable
