# Supplementary material for: Lipid lowering therapy patterns and the risk of cardiovascular events in the 1-year after acute myocardial infarction in United Arab Emirates
Source: PLoS One. 2022 Sep 2;17(9):e0268709. doi: 10.1371/journal.pone.0268709 (PMC9439245; doi:10.1371/journal.pone.0268709)
Supplement: S2 Table — (DOCX) [file pone.0268709.s006.docx]

S2 Table: Treatment characteristics - 90-day pre-index LLT use

|  | **Patients’ sample for primary objective** | | **Patients’ sample for secondary objective** | |
| --- | --- | --- | --- | --- |
|  | **N=4,595** | | **N=1,740** | |
| **90-day pre-index LLT use (n, %)^*^** |  |  |  |  |
| **Any LLT** | 4,002 | 87.09% | 1,514 | 87.01% |
| PCSK9i | 2 | 0.04% | 1 | 0.06% |
| Statin only | 3,908 | 85.05% | 1,466 | 84.25% |
| High statin intensity | 3,163 | 68.84% | 1,178 | 67.70% |
| Medium statin intensity | 736 | 16.02% | 283 | 16.26% |
| Low statin intensity | 9 | 0.20% | 5 | 0.29% |
| Statin+Ezetimibe | 90 | 1.96% | 48 | 2.76% |
| High statin intensity | 69 | 1.50% | 35 | 2.01% |
| Medium statin intensity | 21 | 0.46% | 13 | 0.75% |
| Low statin intensity | 0 | 0.00% | 0 | 0.00% |
| Ezetimibe only | 4 | 0.09% | 0 | 0.00% |
| No LLT | 593 | 12.91% | 226 | 12.99% |

LLT - Lipid Lowering Therapy; PCSK9i- Protein convertase subtilisin/kexin type 9 inhibitors;

^*^PCSK9i/Statin/Eze use and intensity were measured using a 90-day pre-index look back period
