# Supplementary material for: Lipid lowering therapy patterns and the risk of cardiovascular events in the 1-year after acute myocardial infarction in United Arab Emirates
Source: PLoS One. 2022 Sep 2;17(9):e0268709. doi: 10.1371/journal.pone.0268709 (PMC9439245; doi:10.1371/journal.pone.0268709)
Supplement: S3 Table — (DOCX) [file pone.0268709.s007.docx]

S3 Table. Post-index LLT treatment patterns among all patients discharged with MI

| **Post-index Treatment Characteristics** | **All patients in sample for primary objective** | |
| --- | --- | --- |
|  | **N=4,595** | |
|  | **N** | **%** |
| **3- Month Post-index LLT use^*^ (n, %)** |  |  |
| **Patients with 3-month post index CE** | 4,539 | 100.00% |
| Any LLT | 4,164 | 91.74% |
| PCSK9i | 4 | 0.09% |
| Statin only | 4,031 | 88.81% |
| High statin intensity | 3,297 | 72.64% |
| Medium statin intensity | 730 | 16.08% |
| Low statin intensity | 4 | 0.09% |
| Statin+Ezetimibe | 129 | 2.84% |
| High statin intensity | 108 | 2.38% |
| Medium statin intensity | 21 | 0.46% |
| Low statin intensity | - | 0.00% |
| Ezetimibe only | 4 | 0.09% |
| **6- Month Post-index LLT use^†^ (n, %)** |  |  |
| **Patients with 6-month post index CE** | 4,595 | 100.00% |
| Any LLT | 4,270 | 92.93% |
| PCSK9i | 5 | 0.11% |
| Statin only | 4,100 | 89.23% |
| High statin intensity | 3,233 | 70.36% |
| Medium statin intensity | 862 | 18.76% |
| Low statin intensity | 5 | 0.11% |
| Statin+Ezetimibe | 167 | 3.63% |
| High statin intensity | 137 | 2.98% |
| Medium statin intensity | 29 | 0.63% |
| Low statin intensity | 1 | 0.02% |
| Ezetimibe only | 3 | 0.07% |

CE – Continuous Eligibility; LLT - Lipid Lowering Therapy; PCSK9i - Protein convertase subtilisin/kexin type 9 inhibitors

Note: Index date is included in the post-index period. First prescription in 1- month post-index period and last prescription in 3-month, 6 month or 12 month post-index period were used.

^*^Patients who had at least 1 prescription for LLT during the 3-month post-index period; Denominator was number of patients in sample for primary objective with at least 1 claim during 3-month CE in post-index period

**^†^**Patients who had at least 1 prescription for LLT during the 6-month post-index period; Denominator was number of patients in sample for primary objective with at least 1 claim during 6-month CE in post-index period
