# Supplementary material for: Lipid lowering therapy patterns and the risk of cardiovascular events in the 1-year after acute myocardial infarction in United Arab Emirates
Source: PLoS One. 2022 Sep 2;17(9):e0268709. doi: 10.1371/journal.pone.0268709 (PMC9439245; doi:10.1371/journal.pone.0268709)
Supplement: S4 Table — (DOCX) [file pone.0268709.s008.docx]

S4 Table. Post-index changes in LLT patterns among all patients discharged with MI

|  | **All patients in sample**  **for primary objective**  **N=4,595** | |
| --- | --- | --- |
| **Post-index LLT Changes** | **N** | **%** |
| **Changes in LLT use from first Rx during 3-month pre-index period to last Rx in 1-mon post-index (n, %)** | | |
| **Patients with 1-month post index CE^*^** | 4,425 | 100.00% |
| LLT initiation | 410 | 9.27% |
| No LLT initiation | 152 | 3.44% |
| Discontinuation | 311 | 7.03% |
| Statin Intensified | 405 | 9.15% |
| Statin Lowered | 161 | 3.64% |
| Statin to Eze Augmentation | 16 | 0.36% |
| Eze to Statin Augmentation | 1 | 0.02% |
| Statin switch to Eze | 1 | 0.02% |
| Eze switch to Statin | 1 | 0.02% |
| Statin Same | 2,961 | 66.92% |
| Eze Same | 1 | 0.02% |
| Statin drop off | 1 | 0.02% |
| Switch to PCSK9i | 1 | 0.02% |
| PCSK9i Same | 2 | 0.05% |
| PCSK9i Intensified | 1 | 0.02% |
| **Changes in LLT use from first Rx during 1-mon post-index period to last Rx in 6-mon post-index (n, %)** | | |
| **Patients with 6-month post index CE^b^** | 4,595 | 100.00% |
| LLT initiation | 184 | 4.00% |
| No LLT initiation | 325 | 7.07% |
| Discontinuation | - | 0.00% |
| Statin Intensified | 365 | 7.94% |
| Statin Lowered | 399 | 8.68% |
| Statin to Eze Augmentation | 62 | 1.35% |
| Eze to Statin Augmentation | - | 0.00% |
| Statin switch to Eze | - | 0.00% |
| Eze switch to Statin | 1 | 0.02% |
| Statin Same | 3,252 | 70.77% |
| Eze Same | 2 | 0.04% |
| Statin drop off | - | 0.00% |
| Switch to PCSK9i | 1 | 0.02% |
| PCSK9i Same | 4 | 0.09% |
| PCSK9i Intensified | - | 0.00% |
| **Changes in LLT use from first Rx during 1-mon post-index period to last Rx in 12-mon post-index (n, %)** | | |
| **Patients with 12-month post index CE^c^** | 4,595 | 100.00% |
| LLT initiation | 208 | 4.53% |
| No LLT initiation | 301 | 6.55% |
| Discontinuation | - | 0.00% |
| Statin Intensified | 356 | 7.75% |
| Statin Lowered | 485 | 10.55% |
| Statin to Eze Augmentation | 99 | 2.15% |
| Eze to Statin Augmentation | 1 | 0.02% |
| Statin switch to Eze | - | 0.00% |
| Eze switch to Statin | - | 0.00% |
| Statin Same | 3,135 | 68.23% |
| Eze Same | 2 | 0.04% |
| Statin drop off | - | 0.00% |
| Switch to PCSK9i | 4 | 0.09% |
| PCSK9i Same | 4 | 0.09% |
| PCSK9i Intensified | - | 100.00% |

EZE- ezetimibe; LLT - lipid Lowering Therapy; PCSK9i - Protein convertase subtilisin/kexin type 9 inhibitors; Rx - prescription

^*^Patients with 1-month post index CE. Patients with at least one claim for any service (i.e., Drug, Procedures, consultation etc. in any market CVD or non-CVD) in 1-month post index period, these patients were selected and the change in LLT use is reported.
